# Multiscale Entropy of Electroencephalogram as a Potential Predictor for the Prognosis of Neonatal Seizures

**DOI:** 10.1371/journal.pone.0144732

**Published:** 2015-12-11

**Authors:** Wen-Yu Lu, Jyun-Yu Chen, Chi-Feng Chang, Wen-Chin Weng, Wang-Tso Lee, Jiann-Shing Shieh

**Affiliations:** 1 Department of Pediatrics, New Taipei City Hospital, Taipei, Taiwan; 2 Department of Pediatrics, National Taiwan University Hospital, Taipei, Taiwan; 3 Department of Mechanical Engineering, Yuan Ze University, Taoyuan, Taiwan; 4 Clinical Center for Neuroscience and Behavior, National Taiwan University Hospital, Taipei, Taiwan; 5 Graduate Institute of Brain and Mind Sciences, National Taiwan University, Taipei, Taiwan; 6 Innovation Center for Big Data and Digital Convergence, Yuan Ze University, Taoyuan, Taiwan; University of Toronto, CANADA

## Abstract

**Objective:**

Increasing animal studies supported the harmful effects of prolonged or frequent neonatal seizures in developing brain, including increased risk of later epilepsy. Various nonlinear analytic measures had been applied to investigate the change of brain complexity with age. This study focuses on clarifying the relationship between later epilepsy and the changes of electroencephalogram (EEG) complexity in neonatal seizures.

**Methods:**

EEG signals from 19 channels of the whole brain from 32 neonates below 2 months old were acquired. The neonates were classified into 3 groups: 9 were normal controls, 9 were neonatal seizures without later epilepsy, and 14 were neonatal seizures with later epilepsy. Sample entropy (SamEn), multiscale entropy (MSE) and complexity index (CI) were analyzed.

**Results:**

Although there was no significant change in SamEn, the CI values showed significantly decreased over Channels C3, C4, and Cz in patients with neonatal seizures and later epilepsy compared with control group. More multifocal epileptiform discharges in EEG, more abnormal neuroimaging findings, and higher incidence of future developmental delay were noted in the group with later epilepsy.

**Conclusions:**

Decreased MSE and CI values in patients with neonatal seizures and later epilepsy may reflect the mixed effects of acute insults, underlying brain immaturity, and prolonged seizures-related injuries. The analysis of MSE and CI can therefore provide a quantifiable and accurate way to decrypt the mystery of neonatal seizures, and could be a promising predictor.

## Introduction

The incidence of neonatal seizures accounts for about 1–3 per 1000 live births, and eighty percent of cases with neonatal seizures occurs in the first week of life [1]. The immature brain is prone to seizures due to extrinsic and intrinsic factors [2]. The extrinsic factors include various prenatal, perinatal, or postnatal insults, like hypoxic-ischemic episodes, intracranial hemorrhage, infections, congenital brain anomalies, or inborn errors of metabolism. The present dilemma in the management of neonatal seizures is on whether we should treat and how long we should treat. Both clinical and electrographic neonatal seizures may be associated with neurological sequelae (including motor and cognitive deficits) and an increased risk of epilepsy later in life [3]. However, even with increasing knowledge of potentially serious consequences of neonatal seizures, the management remained unchanged over the past decades [3, 4]. Only limited unfavorable predictors of neurological outcome in neonatal seizures had been raised, such as interictal electroencephalographic (EEG) abnormalities [1] and underlying etiologies [4]. Abnormal EEG findings, including burst-suppression patterns and multifocal epileptiform activity, had been shown to be poor prognostic predictors [1, 5]. Postnatal epilepsy tends to develop in neonates with moderate or higher depression on EEG backgrounds [6], which were only identified by EEG morphology and thus could not provide reliable prediction of the development of epilepsy.

Nonlinear dynamical analysis of biological signals had been developed and ameliorated for decades. Multiple measures, including correlation dimension, Lyapunov exponents, and entropy, had been applied to investigate biological signals. Of these methods, multiscale entropy (MSE), extended from sample entropy (SamEn), had been used broadly on analyzing EEG changes, heart rate variability, brain consciousness, and even the signals of electromyogram [7–13]. It is able to evaluate the variability of EEG signals in long-range temporal dynamics, and is more sensitive and accurate than previous methods. The complexity index (CI), calculated by estimating the area under MSE curves, can also demonstrate the structural richness of information over multiple spatial and temporal scales of EEG signals [14]. Therefore, by analyzing the complexity of the EEG signals, the adaptation and functioning of neuronal network in different disease states can be clarified [15].

In the present study, we attempted to analyze and quantify the EEG complexity in neonatal seizures with or without later epilepsy via more accurate nonlinear analytic measures—MSE and CI. We intended to clarify whether there is any difference in EEG complexity based on the analysis of MSE and CI in those neonates with or without later epilepsy.

## Materials and Methods

### Study population characteristics

In this study, all patients and normal controls were enrolled from the Department of Pediatric Neurology in National Taiwan University Children Hospital, which is the major tertiary medical center in Taiwan. EEG was arranged and recorded for at least one hour when there were recurrent seizures in neonates, and was recorded as early as possible before long-term anti-epileptic drugs were used when the patient’s clinical status and vital signs were stabilized. Neonates with status epilepticus or other severe non-neurological conditions like shock were excluded from analysis. Only neonates with recurrent seizures were enrolled in the present study. Totally thirty-two EEG from neonates recorded below 2 months of age were analyzed. Data were divided into three groups: those without neonatal seizures or other significant neurological diseases (Group 1, normal controls), those with seizure episodes but no later epilepsy (Group 2), and those with neonatal seizures and later epilepsy (Group 3). The normal controls had EEG studies due to: (1) suspected apnea with/without bradycardia (5 patients), and (2) occasional myoclonus (4 patients). They recovered without any treatment, and the 24-hour EEG did not reveal any abnormality. The seizure types were classified into tonic, clonic, myoclonic seizures or motor automatisms depending on the findings in EEG. Motor automatism usually presents with chewing, pedaling, or ocular movements, is usually not associated with EEG changes, and therefore is different from true electrographic seizures [16]. The demographic data including gestational age, comorbidity, seizure onset age, and follow-up developmental status were acquired. We defined epilepsy later in lifetime as seizures recurred after 2 months of age. We followed up the patients for at least two years. Because this was a retrospective analysis, the Institutional Review Board of National Taiwan University Hospital had approved the present study waiving informed consent (201306046RINC).

### EEG recording

At our center, we use the full 10–20 system of electrodes for all full-term neonatal EEGs for the comparison of later development. The restricted system of electrode placement was only used in prematurity. Therefore, in the present study, EEG signals were acquired using 19-chanel EEG machine (Nihon Kohden) at Fp1, Fp2, Fz, F3, F4, F7, F8, Cz, C3, C4, Pz, P3, P4, T3, T4, T5, T6, O1, and O2 electrode sites, followed the rule of 10/20 international electrode placement in a referential montage with auricle as reference. The EEG was acquired before long-term anti-epileptic drugs were given when there was no clinical seizures. The background interictal EEG segments without artifacts or electrographic seizures were selected for 1 minute and 40 seconds, in which the patients were in light sleep state. The EEG signals were sampled at 200Hz. According to sampling theory, we can only capture 100Hz signals for sampling frequency 200 Hz. Therefore, total 1 x 10^4^ points acquired in 100 seconds can be used for analysis.

### Multiscale entropy (MSE)

MSE analysis is a method of measuring the complexity of finite length time series. The calculation was based on the benchmark from SamEn analysis, in which only single scale was analyzed, which was then further modified by Costa et al using multiscale analysis method to more accurately apply to the complex physiological time series [9, 10, 15]. Given a one-dimensional discrete time series, {*x*1, *x*2,…, *x*N}, we construct consecutive coarse-grained time series, {y(*τ*)}, determined by the scale factor, *τ*, according to Eq 1 below:

$$y_{j}^{\left(\tau \right)}=\frac{1}{\tau }\Sigma_{i=(j-1)\tau +1}^{j\tau }x_{i},\;1\leq j\leq \frac{N}{\tau }$$ (1)

For scale one, the time series {y^(1)^} is simply the original time series. The length of each coarse-grained time series is equal to the length of the original time series divided by the scale factor, *τ* [15]. Here we consider time series with 2 x 10^4^ points (acquired in 100 seconds) and coarse-grain them up to scale 20, so that the shortest time series has 1000 points. We then calculated an entropy measure for each coarse grained time series plotted as a function of the scale factor τ [17]. SamEn is thus calculated for each time series {y(τ)}. SamEn is a measurement of irregularity of data points. The calculations of SamEn were expressed as follows in Eq 2:

$$\text{SamEn}\left(\text{m},\text{r},\text{N}\right)=\;-\text{ln}\frac{C^{m+1}\left(r\right)}{C^{m}\left(r\right)}$$ (2)

Where $\;C^{m}\left(r\right)={\left(N-m\right)}^{-1}\Sigma_{i}^{N-m}C_{i}^{m}\left(r\right)$

Therein, $\text{d}=\left|x_{i}^{m}-x_{j}^{m}\right|$ denoted the distance between points $x_{i}^{m}$and $x_{j}^{m}$ in the space of dimension m, r is a coefficient of tolerance, SD is a standard deviation of original data, R represents the maximum tolerable distance, and N is the length of the time series.$C_{i}^{m}\left(r\right)$ is the number of all probable pairs (*i*,*j*) with d < R. Various theoretical and clinical applications had proven that SamEn had better statistical validity for m = 1 or 2 and 0.1≦*r*≦0.25 [15]. In the present study, *m* = 2, *r* = 0.15, R = r*SD were applied.

### Complexity index (CI)

CI, distinct from irregularity, has a straight-forward way to present the complexity of cerebral EEG signal [18, 19], and is able to present the structural richness of information over multiple spatial and temporal scales of signals [14]. It is determined by estimating the area under the MSE curve as described previously by Costa. [15]

### Statistical Analysis

Statistical analyses were performed using R version 3.1. The differences in clinical variables in Table 1 were analyzed using the Fisher’s exact test. The results of our evaluations of CI values in isolated channel in the three groups were expressed as the mean ± SD. Wilcoxon rank sum tests were used to compare the differences in the data between Group 1 and Group 2, Group 2 and Group 3, and Group 1 and Group 3. For multiple comparisons, false discovery rates were used to derive the q-value between Group 1 vs Group 3 and Group 2 vs Group 3. All statistical tests were two-tailed with α = 0.05.

**Table 1:** Demographic data.

	Control (Group 1) (n = 9)	Neonatal seizures but no epilepsy (Group 2) (n = 9)	Neonatal seizures with later epilepsy (Group 3) (n = 14)
**Sex: No.(%)**			
F	3(33)	2(22)	11(79)*
M	6(67)	7(78)	3(21)
**Mean gestational age (week**±SD**)**	37.92±1.81	38.62±1.44	38.50±1.53
**Median seizure onset age(days after birth)**	-	6	4.5
**Underlying diseases: No.(%)**			
No	9(100)	2(22)	2(14)
Perinatal hypoxemia or asphyxia	-	2(22)	2(14)
Intracranial hemorrhage	-	-	1(7)
Central nervous system infection	-	1(11)	1(7)
Congenital diaphragmatic hernia	-	1(11)	1(7)
Congenital heart diseases	-	-	2(14)
Suspected pachygyria	-	-	1(7)
Chromosomal anomaly	-	1(11)	4(29)
Sepsis	-	1(11)	-
Neonatal jaundice	-	1(11)	-
**Seizure type: No. (%)**			
Motor automatism	-	4(44.4)	3(21.4)
Tonic	-	-	1(7.14)
Clonic	-	2(22.2)	2(14.3)
Myoclonic	-	-	1(7.14)
Tonic + Clonic	-	1(11.1)	2(14.3)
Motor automatism + Clonic	-	1(11.1)	3(21.4)
Motor automatism + Tonic	-	1(11.1)	1(7.14)
Tonic + Clonic + Motor automatism	-	-	1(7.14)
**Mean EEG timing (PMA**±SD**)**	40.78±2.38	41.78±3.09	42.31±2.49
**EEG finding: No.(%)**			
Negative	9(100)	3(33)	2(14)
Focal discharges	-	5(56)	6(43)
Multifocal discharges	-	-	6(43)*
Cerebral dysfunction	-	1(11)	-
**Abnormal neuroimage: No.(%)**	-	3(33)	11(79)*
**Later developmental delay (%)**	1(11)	1/7(14)	9/11(82)*

SD: standard deviation

*P<0.05 when compared with Group 1 or Group 2.

## Results

Totally 32 neonatal EEG recordings from 32 neonates were retrospectively analyzed (S1 Table). Nine who had no seizure or other neurological diseases in neonatal period were enrolled as Group 1 (normal control). Nine with neonatal seizures but no epilepsy diagnosed later in life were Group 2, and 14 with neonatal seizures and later epilepsy were designated Group 3 (Table 1). There was no difference in gestational age among these 3 groups. However, Group 3 consisted of more females than the other groups. 66.7% (6/9) in Group 2 and 57.1% (8/14) in Group 3 had seizure onset within 1 week of age, which was compatible with previous data that neonatal seizures tend to occur during the first week of life [1]. The motor automatism, clonic seizures, and mixed types were the major seizures in Group 2 and 3 (Table 1). On the other hand, the anti-epileptic drugs used in group 2 and 3 were shown in Table 2. For those without epilepsy (Group 2), the anti-epileptic drugs were used for short term, and were discontinued after stabilization.

**Table 2:** Summary of antiepileptic drugs used in group 2 and group 3.

	Neonatal seizures but no epilepsy (Group 2) (n = 9)	Neonatal seizures with later epilepsy (Group 3) (n = 14)
**AED No.:**		
**0**	2	0
**1**		
Lorazepam	1	0
Phenobarbital	6	7
Levetiracetam	0	1
**2**		
Phenobarbital + Levetiracetam	0	1
Phenobarbital + Vit. B6	0	2
**3**		
Phenobarbital + Phenytoin + Topamax	0	1
**5**		
Lorazepam + Phenobarbital + Phenytoin + Dormicum + Depakine	0	1
**6**		
Phenobarbital + Vit. B6 + Phenytoin + Topamax + Trileptal + Depakine	0	1

Compared to those without later epilepsy, more multifocal epileptiform discharges in EEG were seen in patients with later epilepsy (Group 3). Patients with later epilepsy also had more neuroimaging abnormalities compared to those without later epilepsy (79% v.s. 33%). The major neuroimaging findings in Group 3 included one with white matter change, one with intracranial hemorrhage, one with pachygyria, one with encephalomalacia, and five with parenchymal signal changes. In contrast, the major neuroimaging abnormalities in patients without later epilepsy (Group 2) included two with intracranial hemorrhage and one with encephalomalacia. In follow-up, the neurological outcome was also different in Group 2 and 3. The patients with neonatal seizures and later epilepsy (Group 3) showed significant higher incidence of developmental delay later in life (Table 1).

**Fig 1:**
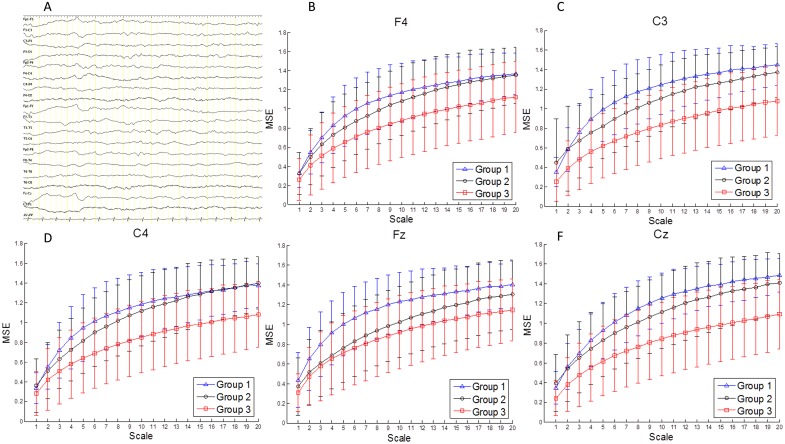
Demonstrated channels showing the changes of multiscale entropy and complexity index (CI) in three groups calculated from conventional EEG in sleep state (A). The CI was lower in Group 3 compared with Group 1 or Group 2 (B-F). However, there was no difference in Group 1 and Group 2. Group 1(Control): those without neonatal seizures; Group 2: those with neonatal seizures but no later epilepsy; and Group 3: those with neonatal seizures and later epilepsy.

To investigate the potential role of EEG complexity in evaluation of neurological outcome in neonates, including the development of later epilepsy, the MSE and CI of acquired EEG signals were analyzed. We found that the MSE revealed lower values in most scales in neonates with later epilepsy compared to normal controls and those without later epilepsy (Fig 1). In contrast, SamEn did not reveal any difference in Group 1 to 3, indicating MSE is more sensitive and accurate, which provides more information and functional change of brain.

When calculating the CI, we found that the CI values in normal controls were higher in central areas (Table 3). The CI values in C3, Cz and Fz were significantly higher than that in F7 (P<0.05) and CI in C4 also tended to higher than that in F7 (P = 0.054), which may indicate the spatio-temporal differences in human brain maturation. The CI values in Group 1 were also higher than those in Group 3. When compared with Group 1 (control group), they showed significant decrease of CI values in Group 3 over Channels C3, C4, and Cz (P = 0.014, 0.049, and 0.010, respectively), and borderline decrease in Channel Fz (P = 0.053). Compared with Group 2, the CI values also showed borderline decrease over Channel Cz in Group 3 (P = 0.058) (Table 3). For multiple comparisons, false discovery rates were used to derive the q-value between Group 1 vs. Group 3 and Group 2 vs. Group 3. We found that q-value was 0.038 in Channels C3 and Cz, and 0.059 in Channels C4 and Fz when comparing Group 3 with Group 1. When comparing Group 3 with Group 2, the CI values also tends to be decreased in C3, C4, and Cz (q-value: 0.066, 0.067, and 0.060, respectively). It indicated that the major differences of CI were also in Channels C3, C4, Cz, and Fz. However, there were no significant differences in CI values between Group 1 and Group 2, indicating that there was no significant decrease of CI values in neonates without later epilepsy compared with normal controls.

**Table 3:** Summary of complexity index (CI).

	Control	With neonatal seizure but	With neonatal seizure and
	(Group 1)	no epilepsy (Group 2)	later epilepsy (Group 3)
**F3**	20.3±5.38	19.47±5.23	16.37±7.90
**C3**	22.28±5.51	20.15±6.55	15.28±6.54 * ^a^
**P3**	19.82±4.01	19.75±7.23	16.92±7.11
**O1**	18.60±5.17	17.21±5.55	15.27±7.36
**F4**	20.96±5.49	19.64±6.64	16.02±7.20
**C4**	21.17±5.74	20.17±6.45	15.55±6.71 * ^b^
**P4**	20.96±4.52	19.52±7.19	17.02±6.61
**O2**	19.14±4.36	18.31±5.85	15.54±6.51
**T3**	17.94±6.25	17.37±5.98	15.82±7.64
**T10**	19.41±6.03	17.47±6.13	16.19±7.67
**T4**	20.24±4.80	18.47±6.70	16.26±6.91
**T6**	18.87±4.97	16.71±5.25	15.69±8.27
**Fp1**	19.41±5.59	18.35±8.35	16.09±7.71
**F7**	16.18±5.70	19.93±8.25	17.67±6.74
**Fz**	22.03±5.51	18.80±7.07	16.83±6.77 ** ^b^
**Cz**	22.20±4.57	20.35±7.06	15.33±6.94 * ^a^, ^c^
**Pz**	18.53±5.39	17.54±6.78	14.96±6.73
**Fp2**	20.61±6.34	16.97±7.30	15.34±7.36
**F8**	19.49±7.27	18.78±7.29	16.59±7.08

*P<0.05 when compared with Group 1;

** P = 0.053

^a^ Q value = 0.038 when compared with Group 1.

^b^ Q value = 0.059 when compared with Group 1.

^c^ P = 0.060 when compared with Group 2.

## Discussion

The neonatal period has the greatest incidence of seizures in life span [1, 4]. The immature brain has enhanced vulnerability to seizures possibly due to various factors, including the imbalance of excitatory and inhibitory receptors in early development and the decreased efficacy of inhibitory neurotransmission [20]. Although neonatal seizures may induce neuroprotection via preconditioning mechanisms [21], more harmful effects to immature brain may occur. In animal models, neonatal seizures may impair cognition, increase anxiety and lead to epileptogenesis [4]. Seizures may also induce alterations in synaptic plasticity, and the immature projections and aberrant neuronal circuits may therefore create epileptogenic circuits [22, 23].

Even more and more evidences on the harmful effects of neonatal seizures to immature brains and ongoing seizures may exacerbate brain injury [24], there is no consensus about whether neonatal seizures should be treated [25], and when to stop anti-epileptic medications [26]. Although postnatal epilepsy tends to develop in neonates with moderate or higher depression on EEG backgrounds [6], there is no quantifiable promising predictor for neurological outcome in neonatal seizures.

In the present study, we found that neonates with later epilepsy tended to have lower values in MSE and CI, indicating they can be a potential predictor for neurological outcome in neonatal seizures. Several entropy measures have been applied in investigating biological signals, such as approximate entropy [27], SamEn [8, 28], and maximum likelihood entropy [29]. In our study, SamEn in EEG did not show any difference in different groups of patients. In contrast, MSE, a modification of SamEn analysis, revealed the dynamic changes of EEG information and can be used to differentiate neonates with or without later epilepsy [8].

In our study, we also found that the EEG complexity over bilateral central and mid-central areas were significantly lower in patients with later epilepsy compared with normal controls. The EEG complexity tends to increase with neurodevelopment and maturation as indicated by post-menstrual age and birth status (premature or full-term) [30–32]. With brain maturation and development, there is increased synchronization of brain signals up to alpha rhythm, and desynchronization of brain signals in the higher beta to lower gamma rhythm [33], leading to increased complexity of EEG. Because neonates with later epilepsy tend to have higher incidence of abnormal neuroimaging and later developmental delay, the lower complexity in brain signals in neonates with later epilepsy may result from the mixed effects of acute insults, underlying brain immaturity, anti-epileptic drugs use, and prolonged seizures-related injuries. These factors may lead to interruption of brain maturation and abnormal synaptogenesis, increasing the incidence of future epileptogenicity. Furthermore, spatio-temporal differences in human brain maturation may also contribute to the significantly lower values of CI in central areas in children with later epilepsy compared with normal controls. The myelination and morphological differentiation of sulci begin in the central region, and progress in an occipito-rostral direction [34], which may lead to higher CI in central areas. The formation of sulci is possibly related to the viscoelastic tensions from white matter fibers connecting cortical areas, and thus may involve neuronal migration and cortico-cortical connections [34]. The possible brain immaturity and harmful effect of neonatal seizures may therefore affect brain development resulting in the significantly greater difference in complexity over bilateral central areas in patients with later epilepsy.

About the significant gender difference in Group 3 (more females in group 3), the result seemed to be contrary to current knowledge, which suggests sexual differentiation of the developing brains tends to confer a greater susceptibility to seizures in neonatal males, and may account for the greater damage and poorer outcome in males suffering after early life seizures [35, 36]. However, due to the complicated underlying diseases in group 3 and quite small numbers of our study, further larger prospective studies are needed to clarify the difference.

There are some limitations of the present study. This is a retrospective study with groups differed on the types of brain damage, and this may contribute to the difference in the development of epilepsy. However, some patients in both groups have the same underlying etiologies, indicating underlying brain damage is not singly responsible for the low MSE and CI in neonates with later epilepsy. There was also significant gender difference in the present study. Nonetheless, the influence of sex on the maturation of entropy remains unknown, and gender difference can’t be explained based on the gender difference in maturation of entropy.

In conclusion, the analysis of MSE and CI provide us a quantifiable tool to evaluate the mixed effects of neonatal seizures on brain, and can be a potential predictor to clarify who will be more likely to have later epilepsy. Owing to retrospective character in the present study, further larger prospective studies comprising serial EEG data are needed in the future to clarify the role of MSE and CI in neonatal seizures and later epilepsy.

## Supporting Information

S1 Table Patients’ profile for Groups 2 and 3. (DOC)
