# Supplementary material for: Multiscale Entropy of Electroencephalogram as a Potential Predictor for the Prognosis of Neonatal Seizures
Source: PLoS One. 2015 Dec 11;10(12):e0144732. doi: 10.1371/journal.pone.0144732 (PMC4676749; doi:10.1371/journal.pone.0144732)
Supplement: S1 Table — (DOC) [file pone.0144732.s001.doc]

| **Supplementary table: Patients’ profile for Groups 2 and 3** | | | | | | | | | | |
| --- | --- | --- | --- | --- | --- | --- | --- | --- | --- | --- |
|  | **Sex** | **Gestational age** | **Seizure onset age(day)** | **Underlying disease** | **Seizure type** | **EEG findings** | | **Neuroimage**  **findings** | **Outcome** | |
| **Group 2** | | | | | | | | | | |
| 1 | M | 37 | 68 | Chromosomal anomaly | Motor automatism | | Negative | Ventriculomegaly | Developmental delay + Autism | |
| 2 | M | 40 | 1 | Perinatal asphyxia | Motor automatism | | Focal spikes | Negative | Normal | |
| 3 | M | 36 | 7 | Severe neonatal jaundice | Motor automatism | | Focal spikes | Negative | Normal | |
| 4 | M | 41 | 5 | No | Tonic - Clonic | | Focal spikes | Negative | Normal | |
| 5 | M | 38 | 6 | Perinatal asphyxia | Clonic | | Focal spikes | Suspect petechial hemorrhage | Developmental delay | |
| 6 | F | 38 | 1 | Congenital diaphragmatic hernia | Clonic | | Negative | Negative | Normal | |
| 7 | M | 38 | 8 | Aseptic meningitis | Motor automatism + Tonic | | Cerebral dysfunction | Negative | Normal | |
| 8 | M | 39 | 0 | Sepsis | Motor automatism + Clonic | | Focal spikes | Suspected old insults | Normal | |
| 9 | F | 38 | 44 | No | Motor automatism | | Negative | Negative | Normal | |
| **Group 3** | | | | | | | | | | |
| 1 | F | 39 | 4 | Pachygyria | Clonic | | Focal spikes | pachygyria | | Developmental delay |
| 2 | F | 40 | 2 | Folic acid responsive epilepsy | Tonic | | Multifocal spikes | Normal | | Developmental delay |
| 3 | M | 40 | 23 | Intracranial hemorrhage | Motor automatism + Tonic | | Multifocal spikes | Acute subdural hemorrhage | | Developmental delay |
| 4 | F | 39 | 21 | Congenital heart disease | Motor automatism + Clonic | | Negative | Macrocystic encephalomalacia | | Developmental delay |
| 5 | F | 35 | 24 | Incontinentia pigmenti | Tonic + Clonic | | Spikes over bilateral temporal areas | Leukodystrophy over left hemisphere and right parietal area | | Developmental delay |
| 6 | F | 39 | 24 | Congenital heart disease | Motor automatism | | Negative | Negative | | Loss of follow up |
| 7 | F | 37 | 1 | Perinatal asphyxia | Motor automatism + Clonic | | Multifocal spikes | High-signal foci near both lateral thalami, both basal ganglia, superior corona radiata | | Developmental delay |
| 8 | M | 39^+2^ | 3 | No | Motor automatism | | Focal spikes | Negative | | Developmental delay |
| 9 | F | 39 | 2 | Streptococcus Bovis meningitis | Tonic + Clonic + Motor automatism | | Focal spikes | Negative | | Developmental delay |
| 10 | F | 39 | 5 | Incontinentia pigmenti | Tonic + Clonic | | Multifocal spikes | Acute focal ischemic changes | | Normal |
| 11 | F | 38 | 1 | Incontinentia pigmenti | Motor automatism | | Focal spikes | Negative | | Developmental delay |
| 12 | M | 39 | 7 | - | Myoclonic | | Multifocal spikes | Negative | | Developmental delay |
| 13 | F | 36 | 30 | Congenital diaphragmatic hernia | Clonic | | Multifocal spikes | Ventriculomegaly | | Developmental delay at 1 year 6 months |
| 14 | F | 37 | 1 | Perinatal asphyxia | Motor automatism + Clonic | | Focal spikes | Increased signal intensity in left lateral thalamus | | Normal |
